# Supplementary material for: Targeting ferroptosis by poly(acrylic) acid coated Mn3O4 nanoparticles alleviates acute liver injury
Source: Nat Commun. 2023 Nov 21;14:7598. doi: 10.1038/s41467-023-43308-w (PMC10663555; doi:10.1038/s41467-023-43308-w)
Supplement: Supplementary file 1 — Supplementary Information [file 41467_2023_43308_MOESM1_ESM.pdf]

Supplementary Information for

Targeting Ferroptosis by Poly(acrylic) Acid Coated Mn_3O_4 Nanoparticles Alleviates Acute Liver Injury

Xinyi Shan[a,b,#], Jiahuan Li[a,b,#], Jiahao Liu[b,c,d,#], Baoli Feng[a,b], Ting Zhang[a,b], Qian Liu[a,b], Huixin Ma[b,c], Honghong Wu[b,c,e,f,]*, Hao Wu[a,b,]*

[a]State Key Laboratory of Agricultural Microbiology, College of Veterinary Medicine, Huazhong Agricultural University, Wuhan 430070, China

[b]Hubei Hongshan Laboratory, Wuhan 430070, China

[c]MOA Key Laboratory of Crop Ecophysiology and Farming System in the Middle Reaches of the Yangtze River, College of Plant Science & Technology, Huazhong Agricultural University, Wuhan 430070, China

[d]College of Agriculture, Tarim University, Alar 843300, China

[e]Shenzhen Institute of Nutrition and Health, Huazhong Agricultural University, Wuhan 430070, China

[f]Shenzhen Branch, Guangdong Laboratory for Lingnan Modern Agriculture, Genome Analysis Laboratory of the Ministry of Agriculture, Agricultural Genomics Institute at Shenzhen, Chinese Academy of Agricultural Sciences, Shenzhen 518120, China

[#]These authors contribute equally

*Corresponding authors

E-mail address:

Honghong Wu, honghong.wu@mail.hzau.edu.cn

Hao Wu, whao.1988@mail.hzau.edu.cn

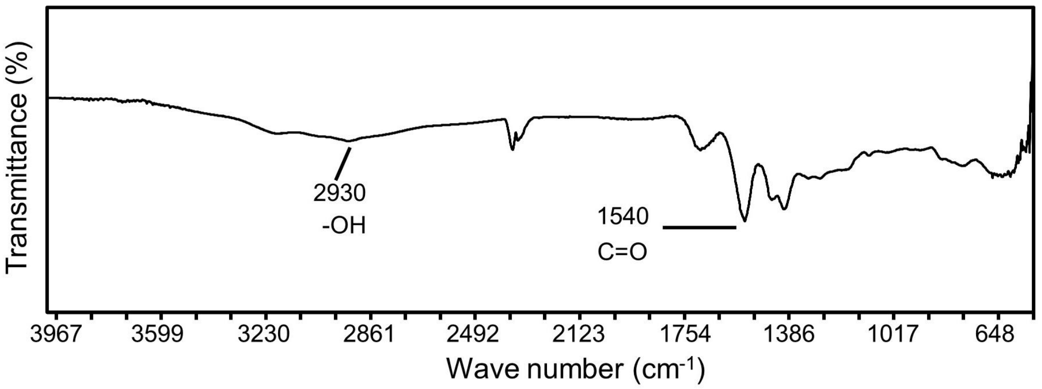

Supplementary Fig. 1: FTIR analysis of PMO. The noted groups are -OH and C=O.

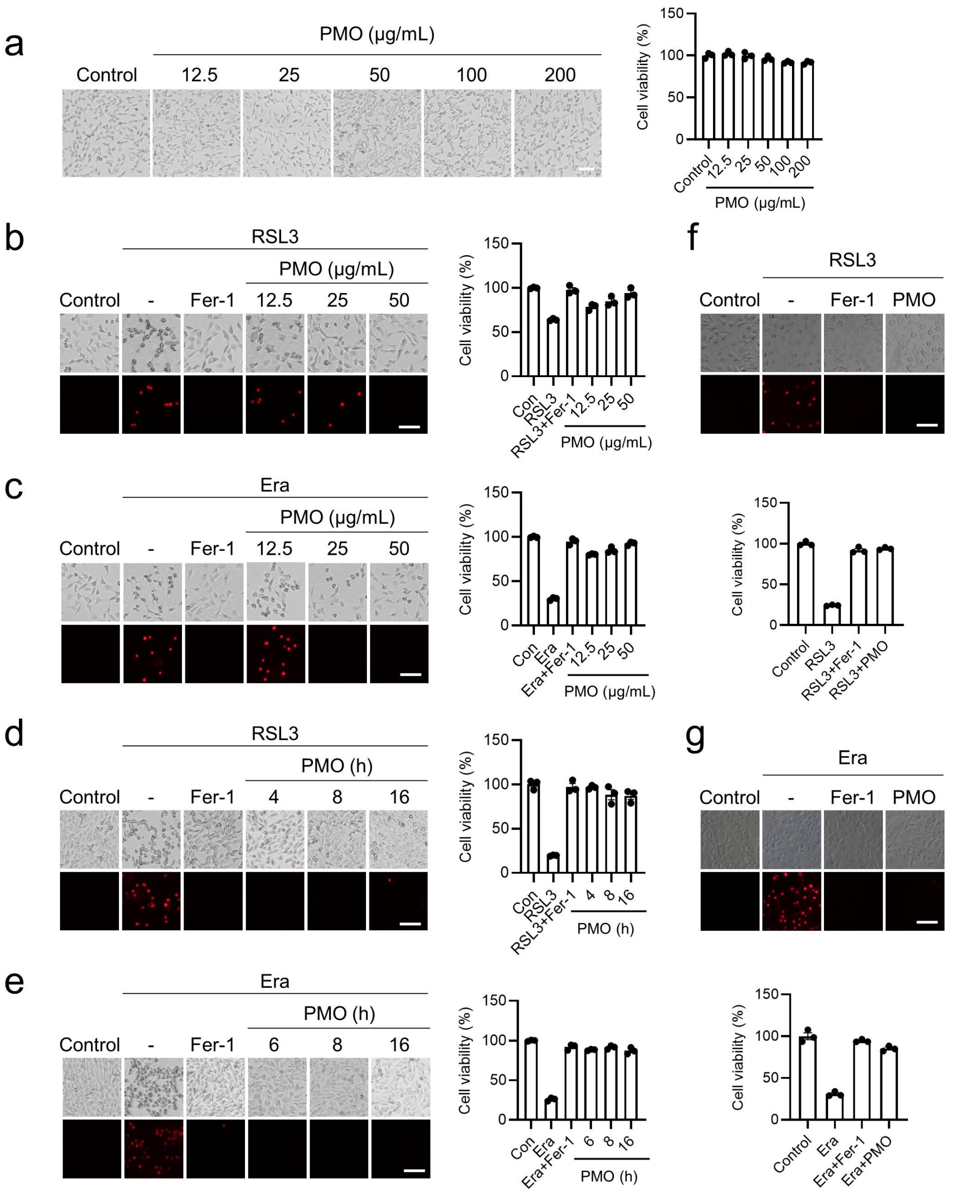

Supplementary Fig. 2: PMO inhibit ferroptosis. a MEFs were incubated with the indicated dose of PMO for 6 h. The representative microscopy images and relative cell viability measured by CCK-8 assay suggested no notable cytotoxicity of PMO. Scale bar = 100 μm. **b**, **c** MEFs were treated with 1 μM RSL3 (**b**) or 10 μM Era (**c**), with or without the indicated dose of PMO or 10 μM Fer-1. Cell death was visualized by PI staining. Scale bar = 100 μm. The relative cell viability was measured by CCK-8 assay. **d**, **e** MEFs were treated with 1 μM RSL3 (**d**) or 10 μM Era (**e**), with or without 50 μg/mL PMO or 10 μM Fer-1 for the indicated time. Cell death was visualized by PI staining. Scale bar = 100 μm. The relative cell viability was measured by CCK-8 assay. **f**, **g** HT1080 cells were treated with 5 μM RSL3 (**f**) or 10 μM Era (**g**), with or without 50 μg/mL PMO or 10 μM Fer-1. Cell death was visualized by PI staining. Scale bar = 100 μm. The relative cell viability was measured by CCK-8 assay. For statistical analysis, data represent mean ± SEM. n = 3 samples in **a-g**. Source data are provided as a Source Data file.

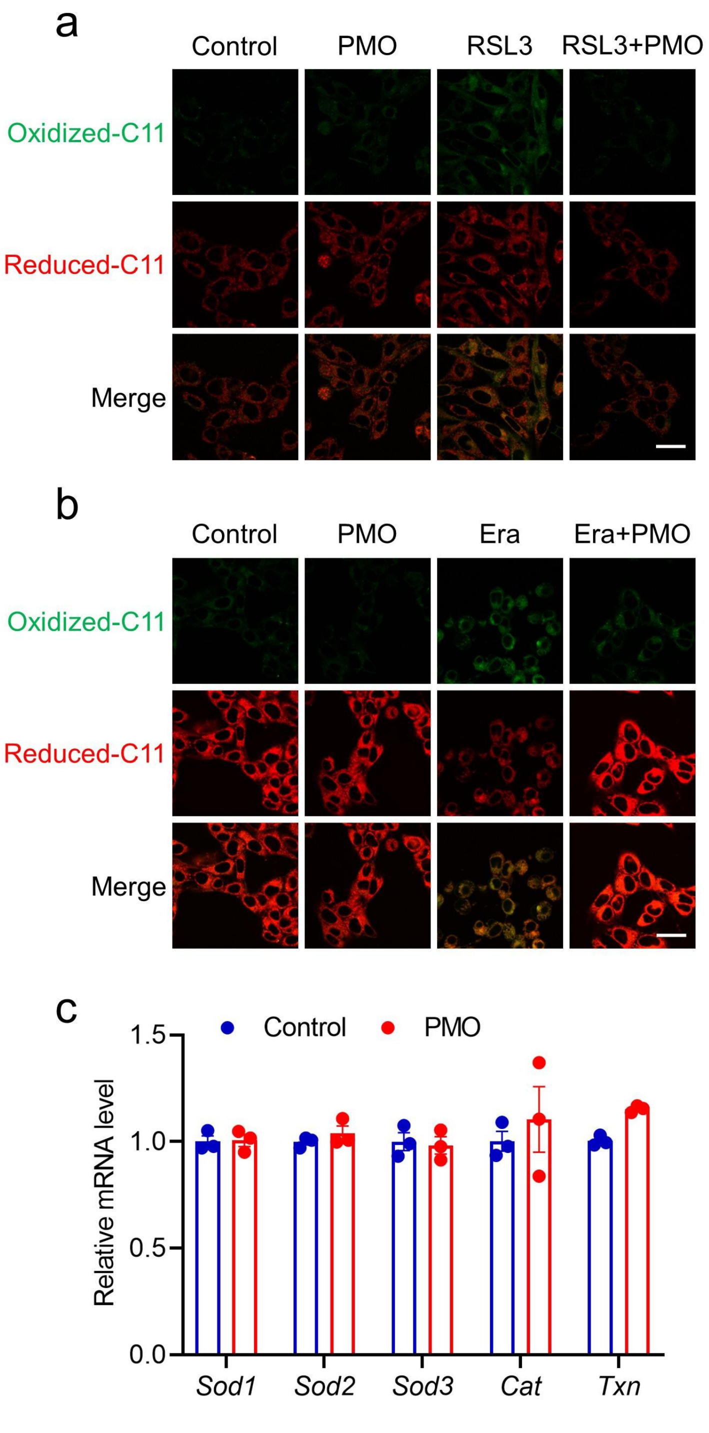

Supplementary Fig. 3: PMO suppress lipid peroxidation. **a**, **b** MEFs were treated with 1 μM RSL3 (**a**) or 10 μM Era (**b**), with or without 50 μg/mL PMO. Lipid peroxidation was analysed by BODIPY 581/591-C11 staining followed by confocal microscopy imaging. The staining was repeated three times independently with similar results. Scale bar = 30 μm. **c** MEFs were treated with 50 μg/mL PMO. Real-time PCR was carried out to check the transcription of the indicated antioxidant genes. For statistical analysis, data represent mean ± SEM. n = 3 samples in **c**. Source data are provided as a Source Data file.

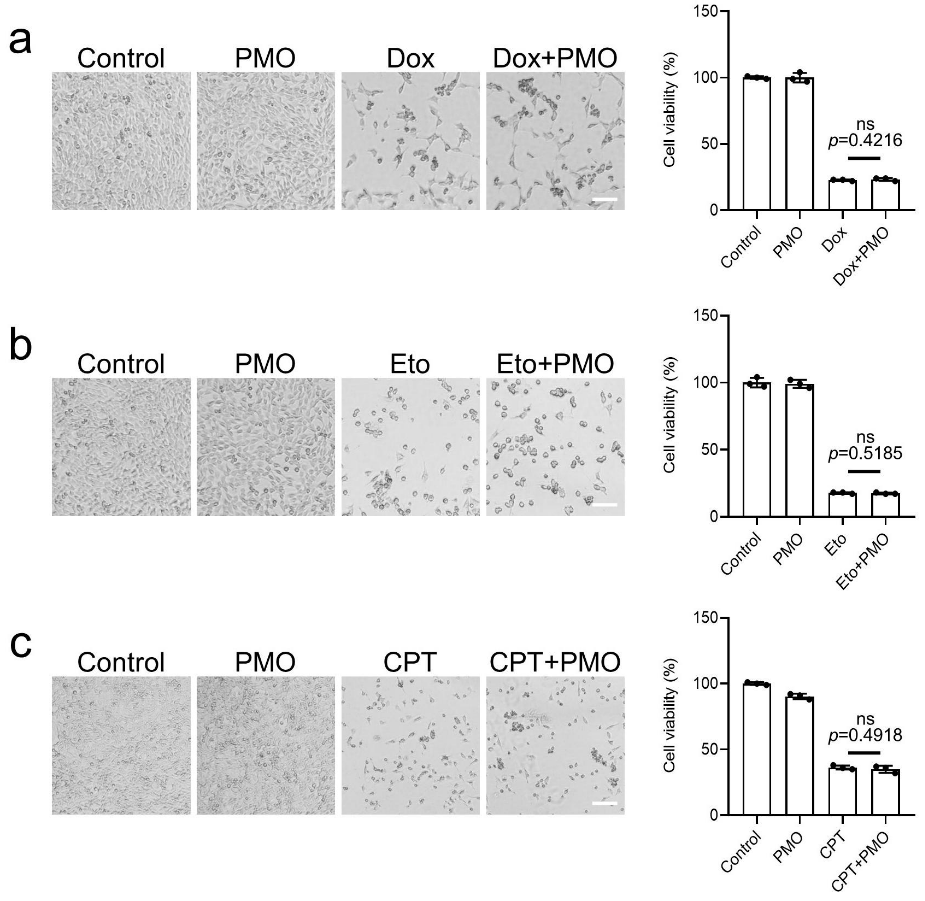

Supplementary Fig. 4: PMO fail to inhibit apoptosis. a-c MEFs were treated with 10 µM Dox (**a**), 50 µM Eto (**b**), or 1 µM CPT (**c**), with or without 50 µg/mL PMO. Cell death was visualized by microscopy imaging. Scale bar = 100 µm. The relative cell viability was measured by CCK-8 assay and shown in the right histograms. For statistical analysis, data represent mean ± SEM. n = 3 samples in **a-c**. ns $P > 0.05$, was determined by two-tailed unpaired Student's t test. Source data are provided as a Source Data file.

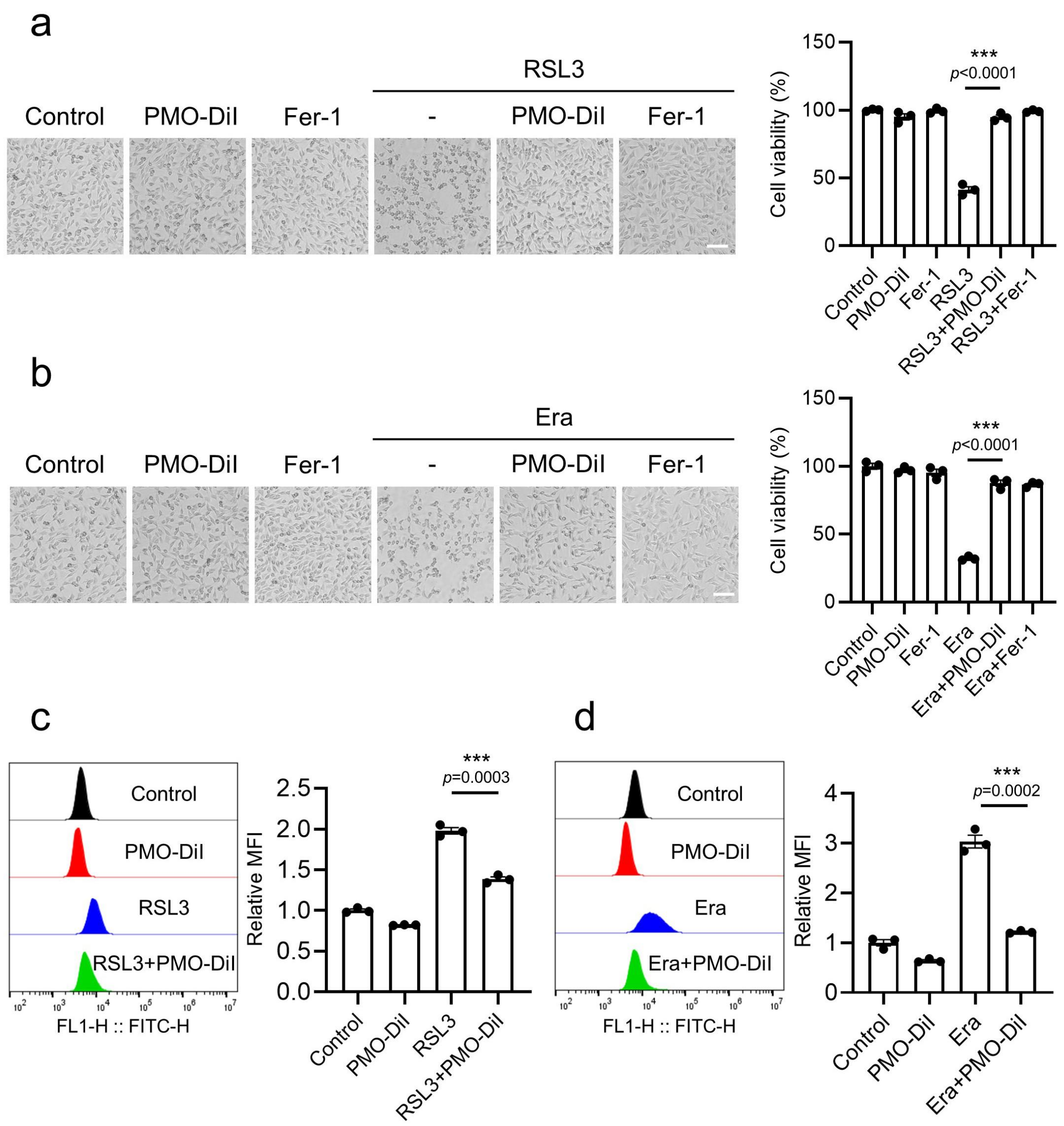

Supplementary Fig. 5: PMO-DiI inhibit ferroptosis and reduce lipid peroxidation. a, **b** MEFs were treated with 1 µM RSL3 (**a**) or 10 µM Era (**b**), with or without 20 µg/mL PMO-DiI or 10 µM Fer-1. Cell death was visualized by microscopy imaging. Scale bar = 100 µm. The relative cell viability was measured by CCK-8 assay. **c**, **d** Cells were treated as in (**a**) and (**b**). Lipid peroxidation was measured by BODIPY 581/591-C11 staining followed by flow cytometry analysis. For statistical analysis, data represent mean ± SEM. n = 3 samples in **a-d**. *** $P < 0.001$, was determined by two-tailed unpaired Student's t test. Source data are provided as a Source Data file.

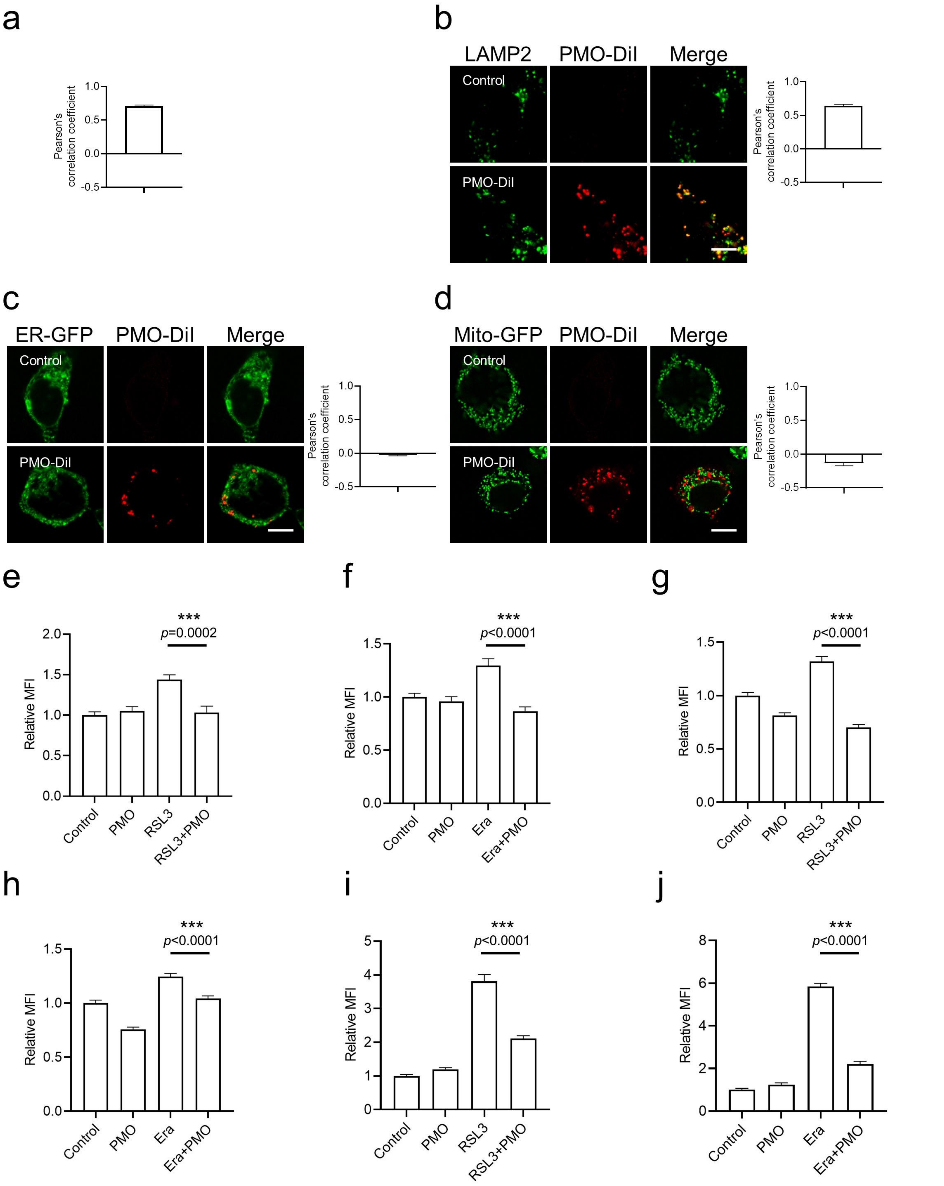

Supplementary Fig. 6: PMO-DiI mainly reside in lysosomes. a Histogram showing Pearson's correlation coefficient between LysoTracker-Green and PMO-DiI in Fig. 4a. **b** MEFs were incubated with 20 μg/mL PMO-DiI. The cells were fixed and stained with anti-LAMP2 antibody. Images were captured to show the colocalization of PMO-DiI with LAMP2. The staining was repeated three times independently with similar results. Scale bar = 10 μm. Histogram showing Pearson's correlation coefficient between PMO-DiI and LAMP2. **c**, **d** Cells were transfected with ER-GFP (**c**) or Mito-GFP (**d**). After 24 h of transfection, the cells were incubated with 20 μg/mL PMO-DiI. The representative images were shown. The staining was repeated three times independently with similar results. Scale bar = 10 μm. The corresponding histogram showing Pearson's correlation coefficient. **e-j** The relative fluorescence intensities of lysosomal FTL (**e**, **f**), lysosomal FerroOrange (**g**, **h**) and lysosomal Foma-LPO (**i**, **j**) in Fig. 4f-k were analysed by *ImageJ*. For statistical analysis, data represent mean ± SEM. *** $P < 0.001$, was determined by two-tailed unpaired Student's t test. Source data are provided as a Source Data file.

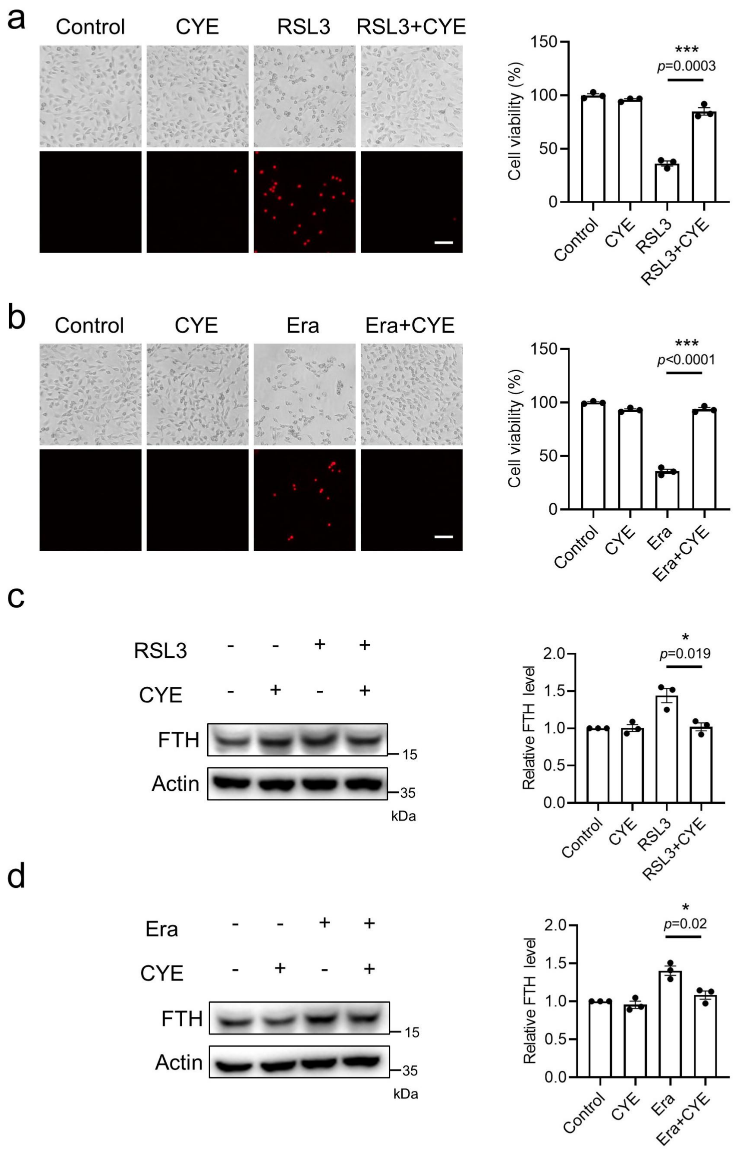

Supplementary Fig. 7: Lysosomal antioxidant cysteamine inhibits ferroptosis and ferritinophagy. a, **b** MEFs were treated with 1 μM RSL3 (**a**) or 10 μM Era (**b**), with or without 10 μM CYE (cysteamine). Cell death was visualized by PI staining. Scale bar = 100 μm. The relative cell viability was measured by CCK-8 assay. **c**, **d** MEFs were treated with 1 μM RSL3 (**c**) or 10 μM Era (**d**), with or without 10 μM CYE. The expressions of FTH were analysed by Western blot. The relative protein levels were quantified. For statistical analysis, data represent mean ± SEM. n = 3 samples in **a-d**. * $P < 0.05$, *** $P < 0.001$, was determined by two-tailed unpaired Student's t test. Source data are provided as a Source Data file.

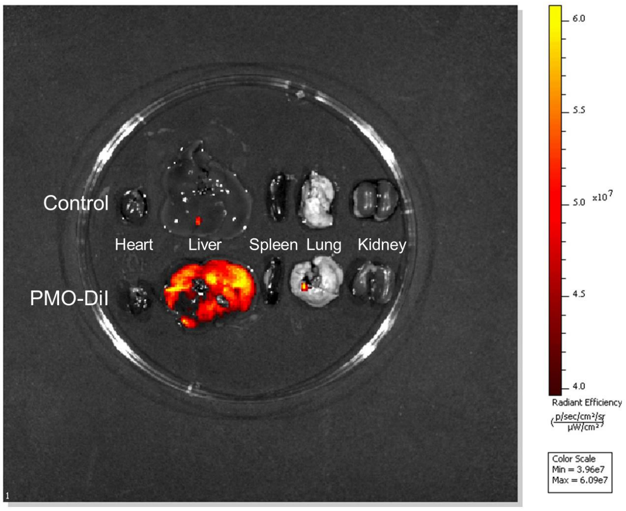

Supplementary Fig. 8: Bioluminescence imaging of PMO-DiI. Mice were injected with PMO-DiI at 20 mg/kg bodyweight. Bioluminescence imaging of the indicated tissues *ex vivo* was conducted 24 h postinjection. This imaging was repeated three times independently with similar results.

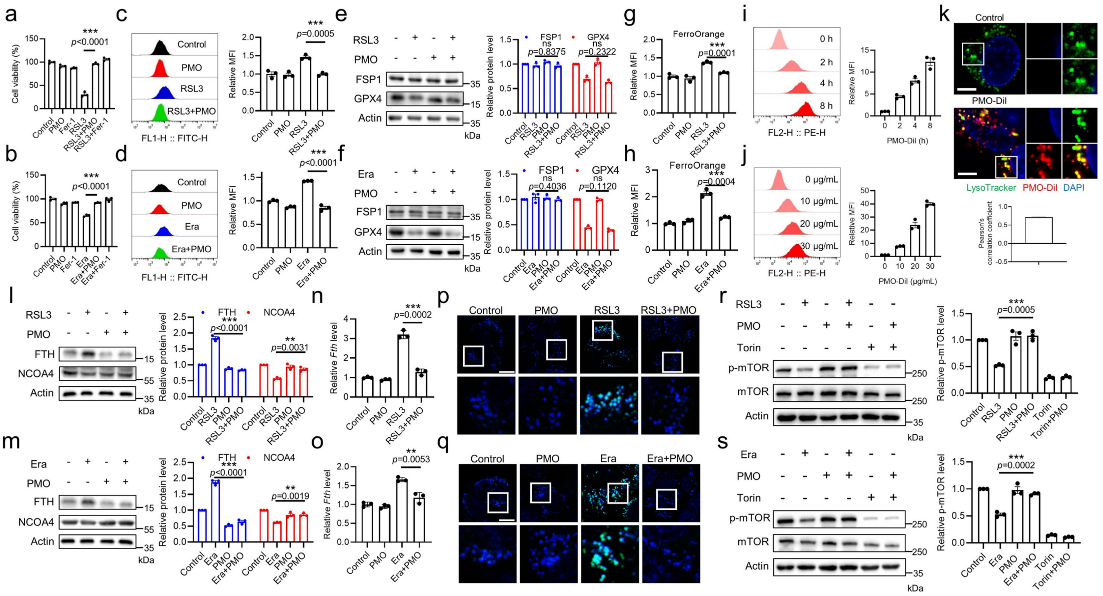

Supplementary Fig. 9: PMO supplementation inhibits ferroptosis in mouse hepatocytes. a, **b** AML12 cells were treated with 5 μM RSL3 for 8 h (**a**), or 10 μM Era for 10 h (**b**), with or without 50 μg/mL PMO or 10 μM Fer-1. The relative cell viability was measured. **c**, **d** AML12 cells were treated as in (**a**) and (**b**). Lipid peroxidation was analysed by BODIPY 581/591-C11 staining. **e**, **f** The expressions of FSP1 and GPX4 were analysed by Western blot. The relative protein levels were quantified. **g**, **h** Intracellular bioactive iron was measured by FerroOrange staining. **i**, **j** Cells were incubated with 20 μg/mL PMO-DiI for the indicated time (**i**), or incubated with the indicated dose of PMO-DiI for 4 h (**j**). Flow cytometry analysis was conducted to show PMO-DiI uptake. **k** Cells were incubated with 20 μg/mL PMO-DiI for 4 h. Lysosomes and nucleus were labeled by LysoTracker and Hoechst, respectively. Images were captured by using a confocal microscope. The staining was repeated three times independently with similar results. Scale bar = 5 μm. Histogram showing Pearson's correlation coefficient between LysoTracker and PMO-DiI. **l**, **m** Cells were treated as in (**a**) and (**b**). The expressions of FTH and NCOA44 were analysed by Western blot. The relative protein levels were quantified. **n**, **o** *Fth* mRNA levels were measured by Real-time PCR. **p**, **q** Cells were treated as in (**a**) and (**b**), then stained with LysoTracker-Blue and Foma-LPO. Images were captured by using a confocal microscope. The staining was repeated three times independently with similar results. Scale bar = 10 μm. **r**, **s** Cells were treated with Torin and RSL3 (**r**) or Era (**s**), with or without 50 μg/mL PMO. The total and phosphorylated mTOR were analysed by Western blot. The phosphorylated mTOR normalized to the total mTOR was shown in the histograms. Data represent mean ± SEM. n = 3 samples in **a-j**, **l-o**, **r-s**. ** $P < 0.01$, *** $P < 0.001$, ns $P > 0.05$, was determined by two-tailed unpaired Student's t test. Source data are provided as a Source Data file.

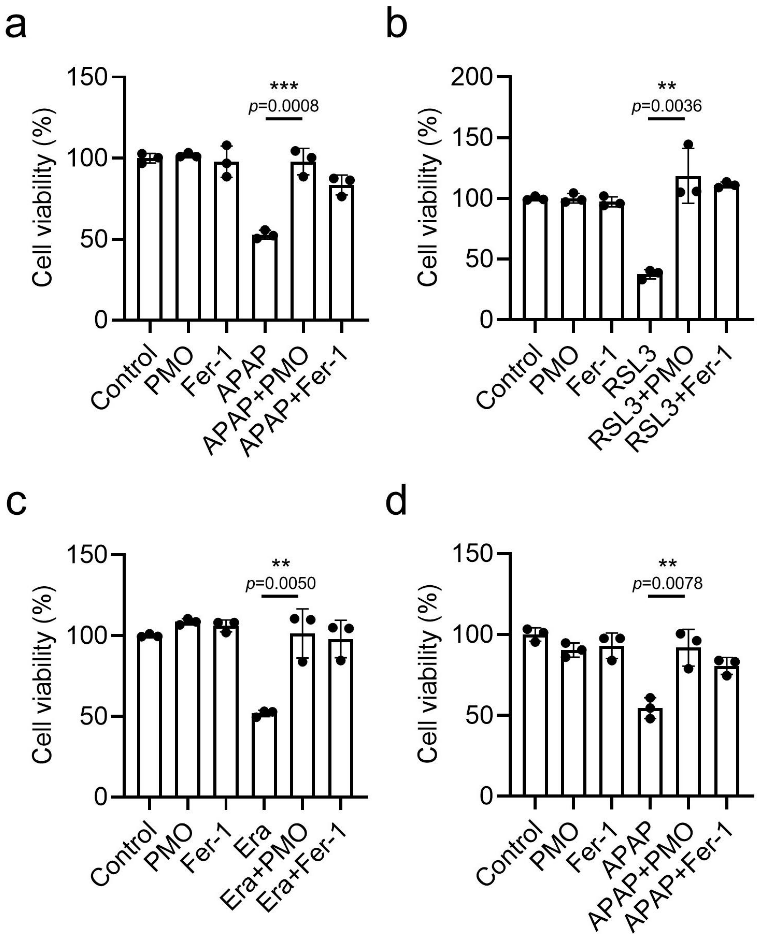

Supplementary Fig. 10: PMO inhibit ferroptosis in hepatocytes. **a** AML12 cells were treated with 20 mM APAP for 24 h, with or without 50 μg/mL PMO or 10 μM Fer-1. The relative cell viability was measured by CCK-8 assay. **b-d** L02 cells were treated with 5 μM RSL3 for 10 h (**b**), 10 μM Era for 16 h (**c**), or 20 mM APAP for 24 h (**d**), with or without 50 μg/mL PMO or 10 μM Fer-1. The relative cell viability was measured by CCK-8 assay. Data represent mean ± SEM. n = 3 samples in **a-d**. ** $P < 0.01$, *** $P < 0.001$, was determined by two-tailed unpaired Student's t test. Source data are provided as a Source Data file.

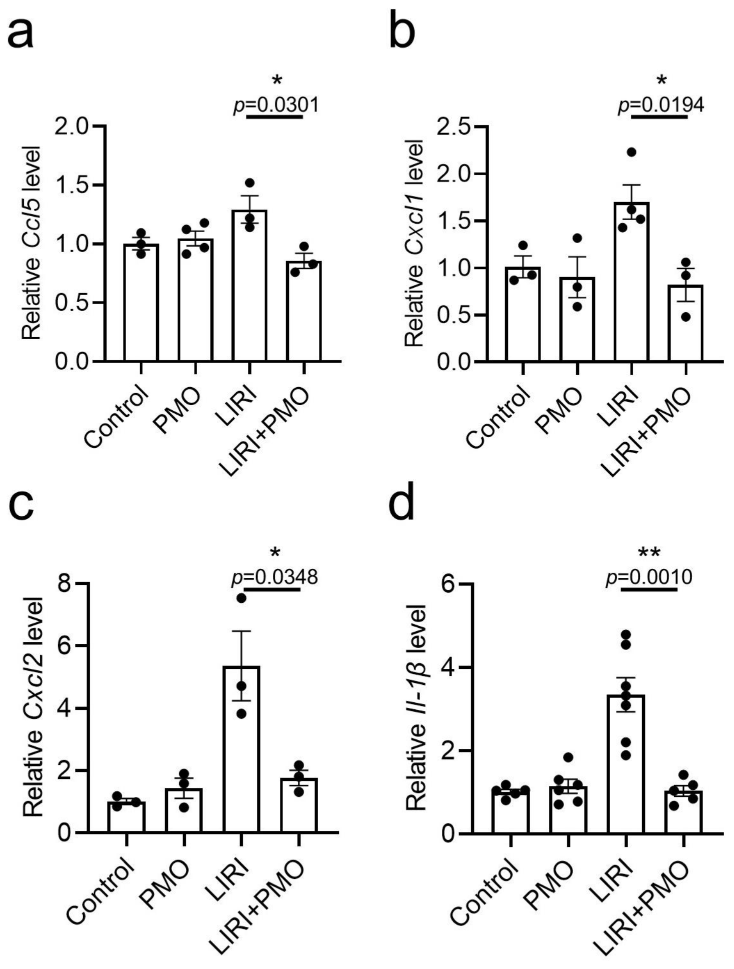

Supplementary Fig. 11: PMO supplementation reduces inflammatory response in LIRI mice. **a-d** Real-time PCR was carried out to check the transcription of *Ccl5* (**a**), *Cxcl1* (**b**), *Cxcl2* (**c**), and *Il-1β* (**d**) in the liver tissues isolated from mice as shown in Fig. 8. For statistical analysis, data represent mean ± SEM. In **a**, n = 3 mice in control group, LIRI group and LIRI+PMO group, n = 4 mice in PMO group. In **b**, n = 3 mice in control group, PMO group and LIRI+PMO group, n = 4 mice in LIRI group. In **c**, n = 3 mice in each group. In **d**, n = 5 mice in control group and LIRI+PMO group, n = 6 mice in PMO group, n = 7 mice in LIRI group. * $P < 0.05$, ** $P < 0.01$, *** $P < 0.001$, was determined by two-tailed unpaired Student's t test. Source data are provided as a Source Data file.

Supplementary Table 1: Antibody list

Antibody	Company	Catalog No.	Clone No. (monoclonal)	Dilution/Application	Validation
GPX4	Abcam	ab125066	EPNCIR144	1:2000/WB	Mouse
4-HNE	Abcam	ab46544		1:200/IHC	Mouse
FSP1	Proteintech	20886-1-AP		1:2000/WB	Human
FTL	Proteintech	10727-1-AP		1:200/IF	Human
Actin	Proteintech	60008-1-Ig	2D4H5	1:10000/WB	Mouse
mTOR	Cell Signaling Technology	2983S	7C10	1:1000/WB	Mouse
p-mTOR	Cell Signaling Technology	5536S	D9C2	1:1000/WB	Mouse
FTH	Cell Signaling Technology	4393S	D1D4	1:2000/WB	Mouse
LAMP2	Santa Cruz Biotechnology	SC-18822	H4B4	1:200/IF	Human
NCOA4	Santa Cruz Biotechnology	SC-373739	C-4	1:1000/WB	Mouse
8-OHdG	Santa Cruz Biotechnology	SC-66036	15A3	1:200/IF	Human
anti-mouse IgG	Cell Signaling Technology	7076S		1:10000/WB	Mouse
anti-rabbit IgG	Cell Signaling Technology	7074S		1:10000/WB	Mouse
goat anti-mouse IgG Alexa Fluor™ 488	Thermo Fisher Scientific	A11029		1:1000/IF	Mouse
goat anti-rabbit IgG Alexa Fluor™ 594	Thermo Fisher Scientific	A11037		1:1000/IF	Mouse

Supplementary Table 2. Primers used in this study

Gene	Forward primer 5’-3’	Reverse primer 5’-3’
β-Actin	CATTGCTGACAGGATGCAGAAGG	TGCTGGAAGGTGGACAGTGAGG
Sod1	TCGTCTTGCTCTCTCTGGTCC	CGAAGTGGATGGTTCCCTGC
Sod2	GGCCAAGGGAGATGTTACAA	GAACCTTGGACTCCCACA
Sod3	GAGAAGATAGGCGACACGCA	GAGAACCAAGCCGGTGATCT
Cat	CACTGACGAGATGGCACACT	ATCGAACGGCAATAGGGGTC
Txn	ATGACTGCCAGGATGTTGCT	TCCTTGTTAGCACCGGAGAA
Fth	GCCGAGAAACTGATGAAGCTGC	GCACACTCCATTGCATTCAGCC
Il-1β	CACTACAGGCTCCGAGATGAACAAC	TGTCGTTGCTTGGTTCTCCTTGTAC
Ccl5	CCTGCTGCTTTGCCTACCTCTC	ACACACTTGGCGGTTCCTTCGA
Cxcl1	GGCTGGGATTCACCTCAAGAACATC	TGAGTGTGGCTATGACTTCGGTTTG
Cxcl2	AACATCCAGAGCTTGAGTGTGACG	GGGCTTCAGGGTCAAGGCAAAC
